# Engineering Extracellular Vesicles as Nanotherapeutics for Regenerative Medicine

**DOI:** 10.3390/biom10010048

**Published:** 2019-12-28

**Authors:** Lalithasri Ramasubramanian, Priyadarsini Kumar, Aijun Wang

**Affiliations:** 1 Surgical Bioengineering Laboratory, Department of Surgery, School of Medicine, University of California–Davis, Sacramento, CA 95817, USApkumar@ucdavis.edu (P.K.); 2 Department of Biomedical Engineering, University of California–Davis, Davis, CA 95616, USA; 3 Institute for Pediatric Regenerative Medicine, Shriners Hospitals for Children–Northern California, Sacramento, CA 95817, USA

**Keywords:** extracellular vesicles, regenerative medicine, biomaterials, stem cells

## Abstract

Long thought of to be vesicles that primarily recycled waste biomolecules from cells, extracellular vesicles (EVs) have now emerged as a new class of nanotherapeutics for regenerative medicine. Recent studies have proven their potential as mediators of cell proliferation, immunomodulation, extracellular matrix organization and angiogenesis, and are currently being used as treatments for a variety of diseases and injuries. They are now being used in combination with a variety of more traditional biomaterials and tissue engineering strategies to stimulate tissue repair and wound healing. However, the clinical translation of EVs has been greatly slowed due to difficulties in EV isolation and purification, as well as their limited yields and functional heterogeneity. Thus, a field of EV engineering has emerged in order to augment the natural properties of EVs and to recapitulate their function in semi-synthetic and synthetic EVs. Here, we have reviewed current technologies and techniques in this growing field of EV engineering while highlighting possible future applications for regenerative medicine.

## 1. Introduction

Regenerative medicine has been a pivotal area of research aimed at healing or replacing damaged tissue. Traditional regenerative strategies have generally seen the use of stem cells and biomaterials as building materials to either replace the lost tissue or promote the regeneration of new tissue [1,2,3]. Recently, a new type of the therapeutic known as exosomes have emerged as a strategy for regenerative medicine [4,5,6]. Exosomes are nanovesicles about 50–150 nm in diameter that are released from almost every type of cell [4,5,7]. They are derived from membrane lipids of parent cells through the fusion of multivesicular bodies with the membrane [7,8]. Exosomes are released to mediate critical cell-to-cell communication by delivering cargo such as proteins, lipids and effector molecules to target cells. In fact, this paracrine cell signaling has been a key area of interest to researchers. Exosomes have been identified to play an important role in several major cell and tissue functions, including cell proliferation and senescence [9,10,11], angiogenesis [12,13,14,15], extracellular matrix support and reorganization [16,17,18] and immunomodulation [19,20,21]. Unsurprisingly, these properties have made exosomes a very attractive therapeutic option for regenerative medicine. 

### 1.1. Exosome Biogenesis

**Figure 1:**
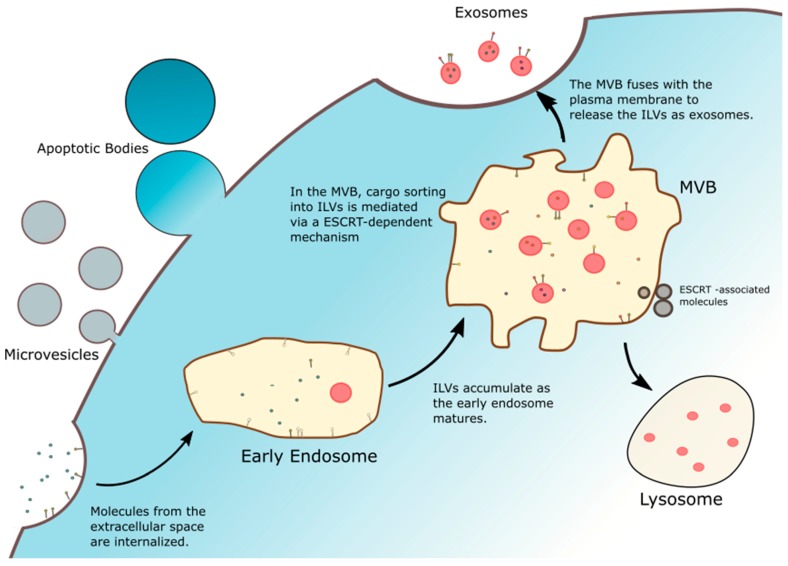
Biogenesis of extracellular vesicles. Microvesicles and apoptotic bodies originate directly from the plasma membrane, while exosomes are derived from the endosomal compartments. intraluminal vesicles (ILVs) accumulate in the multivesicular bodies (MVBs) after early endosome maturation. Proteins, lipids, nucleic acids and other cargo are sequestered within the ILVs through an endosomal sorting complex required for transport (ESCRT)-dependent pathway. Eventually, MVBs fuse with the plasma membrane and release the ILVs into the extracellular space as exosomes.

Membranous vesicles secreted by cells are collectively termed extracellular vesicles (EVs), of which there are three main subtypes: exosomes, microvesicles and apoptotic bodies [8]. These EVs are secreted by most cell types and are ubiquitous in all types of biological fluids, including blood, urine, amniotic fluid, saliva and cerebrospinal fluid [22,23]. Exosomes are the smallest type of EVs (50–150 nm) and, unlike the microvesicles and apoptotic bodies which are directly shed from the plasma membrane, exosomes are released following the fusion of late endosomes and multivesicular bodies with the plasma membrane [22]. Exosome release follows a highly dynamic endocytic pathway (Figure 1). The first step involves the accumulation of intraluminal vesicles (ILVs) as early endosomes mature into late endosomes. These ILVs sort and entrap proteins, lipids and cytosol within these late endosomes, leading to morphological changes that result in multivesicular bodies (MVBs) [7,8,23]. Though in most cases, MVBs fuse with lysosomes for the degradation and recycling of their contents, certain MVBs are decorated with specific proteins and markers that instead ensures their fusion with the plasma membrane and allows the release of their content to the extracellular space and become known as exosomes. This sorting is facilitated by the endosomal sorting complex required for transport (ESCRT), a mechanism which involves about 30 different proteins that help sequester specific biomolecules in the MVBs and guide their release through the plasma membrane as exosomes [8,22,23].

Following release, exosomes are mainly distinguished by the presence of several specific surface markers, namely tetraspanins (CD63, CD9, CD81), which are involved in the sorting of different cargo, the tumor susceptibility gene 101 (TSG) 101 and the apoptosis-linked-gene-2 interacting protein X (ALIX) (proteins associated in ESCRT) [23]. They also express parent cell-specific surface markers as a result of the fusion with the cell plasma membrane [23]. Due to their sub-150nm size, exosomes are mainly visualized using transmission electron microscopy (TEM), where they are observed to have a cup-shaped appearance, though this is more due to the artifacts of the uranyl acetate and methylcellulose fixation process [23]. Cryo-EM, in which samples are cryogenically frozen and vitrified in liquid ethane to prevent structure deformation, revealed that exosomes have a round shape [24]. To further characterize size and concentration, nanoparticle tracking analysis (NTA) is commonly used to calculate exosome diameters by tracking the Brownian movement of individual particles, though there are other methods, such as dynamic light scattering (DLS) and resistive pulse sensing (RPS) [22,25].

### 1.2. Exosome Isolation and Characterization

Exosomes can be isolated from the conditioned media of cultured cells and almost any biological fluid. Current methods of exosome isolation and purification involve ultracentrifugation, precipitation, immunoaffinity and size-based isolation techniques. Ultracentrifugation is currently considered to be the most widespread method by which to obtain exosomes, as it is low cost, and yet it can still isolate particles from large volumes of biological fluids. However, the centrifugation processes may pellet non-exosome contaminants of similar densities [26,27]. Therefore, additional purification steps may be applied, such as a density gradient or ultrafiltration, to further separate out the specific exosome populations. Precipitation is another method of isolating and purifying exosomes. In this method, water-excluding polymers such as polyethylene glycol (PEG) are used to bind water molecules and force less soluble particles such as exosomes out of the aqueous solution [28,29]. This is a relatively simple process and does not require the use of specialized equipment. However, the use of foreign polymers can introduce impurities into the sample that is difficult to remove, and may adversely impact downstream applications [30,31]. A third method, immunoaffinity, relies on the interactions between exosome membrane proteins and their respective antibodies [29] This is a more precise method of capturing specifically exosomes, but is limited in that it cannot be used for large sample volumes. Therefore, it is more commonly used in conjugation with the conventional ultracentrifugation methods where the concentrated pellet is subsequently purified using immunoaffinity. Size exclusion methods include techniques such as size exclusion chromatography and ultrafiltration [26,29]. In both, samples are passed through membranes with predefined size or molecular weight limits, and exosomes are separated from other particles based on these physical characteristics. Size exclusion chromatography and ultrafiltration are relatively simple procedures for exosome isolation. However, size exclusion chromatography requires long columns for ideal size separation, which may be impractical for large scale manufacturing. In ultrafiltration, the use of force that is required to pass the samples through the membrane can potentially damage or deform exosomes. Additionally, exosomes can be lost or otherwise remain adhered to the membrane following this procedure.

As a result of the imprecise and heterogenous nature of exosome isolation, quality control becomes of the utmost importance when developing and establishing clinical-grade exosome treatments. Current good manufacturing practices (cGMPs) can be adopted to standardize exosome isolation procedures by keeping constant the cell sources, as exosomal cargo content can be altered by cell state [26]. Therefore, environmental conditions, such as cell passage number, seeding density or culture conditions, must be kept uniform in order avoid inconsistencies in the quality of the final exosome yield. Purity can be quantitatively assessed by measuring levels of contaminants (e.g., endotoxin, sterility and mycoplasma) through standard clinical tests [32]. Following isolation, current assessments of exosome identity involve an extensive profiling of characteristic surface proteins on both the exosomes and parental cells. Western blotting or flow cytometry can be used to assess the presence of exosome surface proteins (e.g., CD9, CD63, CD81) on the isolated particles, while parental cells are characterized for viability and cell surface marker profiles [33]. Microscopy techniques, such as electron microscopy (EM) and atomic force microscopy (AFM), may be useful for additional morphology and size distribution characterization [32]. Current methods of exosome quantitation mainly rely on the quantification of EV numbers or of EV protein weight [34]. Exosome particle numbers have been commonly calculated through the NTA or other similar concentration measuring instruments and technologies. Exosome total protein content has also been used as a parameter for quantification [32,34]. These quantitative measurements are often used when calculating dosages. Most approaches currently involve administering EVs that are cell equivalent in number (compared to a control treatment group consisting of cells only), as calculated from concentration measurements [31]. Doses can also be administered based on total protein content, though this may be a less than accurate method due to the variation in protein content between individual exosomes [31,35]. Exact clinical doses are dependent on the disease at hand as well as the potency of the exosomes. To assess potency, functional assays can be conducted as a downstream validation process. Otherwise, proteomic or transcriptomic studies can be conducted to directly assess exosomal contents as a way to standardize the bioactive cargo between different batches [32].

### 1.3. Physiological Functions of Exosomes and Implications for Regenerative Medicine

Exosomes were initially thought to be the “trash bags” of the cells, as it was believed that their primary function is to carry waste molecules out of the cells. However, it was soon realized that exosomes carry many important biomolecules, including proteins, nucleic acids and lipids to recipient cells as a way to mediate cell-to-cell communication [6,23,36]. Exosome targeting is still an unclear mechanism, as it is a highly complex process that is dependent upon exosome origin and recipient cell type. Nevertheless, the general mode of targeting relies on receptor-ligand interactions between the surfaces of the target cell and the exosome, respectively. Different types of molecules are involved in these targeting interactions, such as integrins, lipids, lectins, proteoglycans and extracellular matrix components [7,23,24]. Once the exosome is bound to the cell surface, it can affect cell function by either (1) remaining on the cell surface and triggering intracellular signaling pathways or (2) be internalized by the cell through endocytosis, of which both clathrin- and caveolae-dependent and -independent mechanisms have been implicated [24,37,38]. In both cases, exosome contents can be released via fusion and carry out their specific effector functions.

Interestingly, exosome functional properties and the mechanisms by which they mediate cellular processes are also heavily dependent on their cellular origin [5,36,39]. Exosomes derived from different cell types are seen to transfer specific tissue-remodeling molecules and mediate different metabolic pathways to promote the healing and regeneration of varying injured tissue. Mesenchymal stromal cells (MSCs)-derived exosomes, for example, have been shown to have remarkable effects on promoting regeneration [40,41]. Placenta-derived MSCs have been shown to secrete exosomes that play a key role in neuroprotection and immunomodulation [42,43], while exosomes from bone marrow-derived MSCs have been implicated in aiding in bone regeneration [44]. Meanwhile, exosomes derived from endothelial progenitor cells (EPCs) have been shown to deliver microRNAs to promote potent angiogenic effects to induce the vascularization of damaged tissue [12,13,14]. As potent nanotherapeutics, exosomes have the potential to be used in different areas of regenerative medicine, and can be combined with novel engineering strategies for improved functionality and efficiency. However, because of the difficulty in obtaining pure exosome fractions and to consider the possible presence of other vesicular structures, exosomes will henceforth be referred to broadly as extracellular vesicles or EVs in upcoming discussions. This review will focus on the current use of EVs in various regenerative therapies and highlight emerging technologies aimed at augmenting their natural properties with innovative biomedical strategies and systems.

## 2. Biological Stem Cell Derived EVs

### 2.1. Injectable Treatments

The use of EVs is advantageous over cells themselves for multiple reasons. First, cell therapies often suffer from poor post-transplantation cell viability due to the shearing effects when the cells are ejected from a syringe needle [45], and as EVs are not constrained by viability, there is no danger of “death”. Additionally, cell therapies come with a risk of developing tumors or malignant transformations should the transplanted cells become cancerous. EV treatments avoid this disadvantage, as they do not replicate, and instead are recycled after their cargo is delivered. Unwanted host immunogenic responses are additional major hinderances for current cell therapies due to the expression of foreign major histocompatibility complex (MHC) markers that activate host immune responses and may eventually lead to transplant rejection. EVs, however, have immunomodulatory properties that reduce the risk of adverse immune responses while still maintaining regenerative functional properties [46]. EVs also have no need for engraftment, another necessity that has long hindered the effectiveness of previous cell therapies.

Due to their paracrine mechanism of action, EVs have seen great success as injectable therapeutics for neuroprotection, cardioprotection and renoprotection. In a swine model of traumatic brain injury and hemorrhagic shock, animals that received bone marrow MSC-derived EVs via intravenous injection showed significantly lower neurologic impairment and had a faster full neurological recovery to baseline functions [47]. Similarly, intravenous administration of EVs from human embryonic stem cell-derived neural stem cells improved the functional and physical outcomes after thromboembolic stroke in middle-aged mice [48]. Mice that received EV treatment had greater improvements in motor functions and episodic memory while MRI analysis showed significantly less tissue loss. In a parallel porcine model of ischemic stroke, the same neural stem cell-derived EVs showed that intravenous administration of EVs led to significant tissue and functional improvements compared to sham control animals [49]. Pigs that received treatment exhibited normal exploratory behavior and motor activity, suggesting faster recovery, while long term analysis showed a reduced loss of cerebral tissue, decreased brain swelling, and preserved white matter integrity.

The functional properties of EVs are believed to be mainly propagated by their cargo, such as microRNAs. MicroRNAs, or miRNAs, are short strands of noncoding RNA that play crucial roles in mediating gene expression in target cells. EVs carrying specific miRNAs have been preferentially selected for and applied as regenerative therapies. EVs derived from cardiac progenitor cells (CPCs) are highly enriched in miR-210, miR-132 and miR-146a-3p [50]. When injected into infarcted hearts in a rat model, CPC EVs reduce cardiomyocyte apoptosis and enable vascular remodeling, thus exhibiting significant cardioprotective properties through the upregulation or downregulation of miRNA-specific target genes. Proangiogenic properties of MSC-derived EVs are also similarly mediated through the transfer of miRNAs, specifically miR-30b, miR-miR-30c, miR-424 and miR-let-7f, to endothelial cells [15]. Neural regeneration is also seen with the MSC-mediated transfer of miRNA-133b, which regulates neurite outgrowth by interacting with parenchymal cells [51]. Similarly, the delivery of miR-125b by EVs from differentiating neuronal cells could induce the differentiation of human MSCs into neurons [52]. This regulation could have significant regenerative impact in the central nervous system, especially in the aftermath of degenerative or otherwise damaging neurological diseases.

### 2.2. Engineered Scaffolds and Surfaces

Tissue-engineered constructs (i.e., scaffolds) have had great success in recreating structural and functional microenvironments to allow for the regeneration of tissues [53]. As a result, many studies have focused on the use of EVs in combination with engineered scaffolds as a method to improve healing and regeneration. To date, MSC-derived EVs have been widely used to functionalize a variety of scaffold structures due to their proangiogenic properties. Xie et al. coated decalcified bone matrix (DBM) with bone marrow-derived MSC EVs using fibronectin as the immobilizing agent [54]. 

The implanted EV-functionalized scaffolds promoted bone regeneration in a murine model. Significant new bone matrix was observed with twice the average number of CD31-positive vessels within the matrix compared to unmodified scaffolds, confirming the ability of the EVs to promote bone formation and vascularization. EVs can be immobilized on a variety of polymer-based scaffolds, as another study functionalized β-TCP scaffolds with human-induced pluripotent stem cell-derived MSC (hIPS-MSC) EVs [55]. No additional reagent was required to immobilize the EVs on the scaffold; instead, the EVs were simply added to the constructs and were naturally absorbed into the scaffold after incubation. The hIPS-MSC EV-modified scaffolds stimulated osteogenesis in a rat model of a critical-sized calvarial bone defect. The MSC-derived EVs promote osteogenic activity, in that they upregulate the PI3K/Akt signaling pathway by altering over 117 genes. Though MSC-derived EVs have been primarily used due to the natural paracrine signaling that characterize MSC cell function, EVs from other stem cell sources have seen similar success when combined with scaffolds. Poly(lactic-co-glycolic acid) (PLGA/pDA) scaffolds were modified with human adipose-derived stem cell (hASC) EVs using a polydopamine coating to immobilize them on the scaffold [56]. In vivo studies using a murine model of critical-sized calvarial bone defects showed significantly greater bone tissue and mature collagen formation in hASC EV-modified grafts compared to the unmodified grafts. The healing was partially attributed to the ability of the hASC-EVs to recruit host MSCs, which in turn, promoted osteoinductive functions.

Aside from scaffolds, EVs have also been combined with other therapeutic surfaces for wound healing and tissue regeneration. EV-modified sponges and patches have shown to be a promising method of treatment. Shi et al. successfully loaded gingival MSCs (GMSCs), isolated from the gingival lamina propria, onto a chitosan/silk hydrogel sponge as a strategy to promote wound healing in diabetic patients [57]. Much like with scaffolds, EVs could be easily resuspended in phosphate buffered saline (PBS) and added to the hydrogel sponge. The EV-modified sponges were then used as dressings to cover surgically-induced skin wounds in diabetic mice. Quantification of wound size showed that the EV-modified sponges had a significantly greater wound closing effect while immunohistochemical analysis showed higher levels CD34-positive cells, indicating angiogenesis, and greater collagen deposition. EV-modified hydrogel patches have also emerged as a therapeutic for cardiac recovery and regeneration. EVs secreted from induced pluripotent stem cell-derived cardiomyocytes were encapsulated in hydrogel patches [58]. Secreted EVs were enriched in cardiac-specific miRNAs, and when encapsulated in a collagen gelfoam mesh, were sustainably released over 21 days in vitro. Application to rat myocardium revealed similar release profiles and found that EVs promoted arrhythmic recovery, decreased cardiomyocyte apoptosis after infarction, and reduced infarct size and cell hypertrophy. EVs have also been successfully used to modify scaffold-free surfaces to be treated onto monolayer cell sheets. CXCR4-enriched bone marrow–MSC EVs were used to treat MSC cell sheets, which were subsequently implanted into the infarcted area of a rat myocardium [59]. The EVs demonstrated cardioprotective properties in an ischemic injury rat model by upregulating the Akt signaling pathway.

## 3. Semi-Synthetic EVs

With the current surge in EV research and new findings supporting their importance in regenerative medicine, researchers have turned their attention to engineering EVs in order to augment their innate properties to allow for increased therapeutic function.

### 3.1. Drug Delivery

The current gold standard in drug delivery is that of liposomes, synthetic lipid vesicles, which, due to their amphiphilic nature, can encapsulate both hydrophobic and hydrophilic molecules. They are highly biocompatible and can be readily modified for precision medicine, making them a highly popular drug delivery system in current medicine. However, liposomes are quickly cleared from the reticuloendothelial system (RES), making targeted delivery and prolonged treatment difficult. 

Therefore, considering the nanometer-range size, biocompatibility and their natural targeting specificity, EVs have unsurprisingly become a new class of natural nanotherapeutics. The different cargo and cargo loading mechanisms are summarized in Table 1.

#### 3.1.1. Small Molecules

Initial studies focused on the use of EVs, mostly derived from mature cell types, as traditional drug delivery vehicles where drugs or other synthetic therapeutic agents are the cargo of choice. These therapies have met with wide success, as small molecule-loaded EVs have been seen to have great potential in treating inflammation and proinflammatory diseases [60,61], cancer [62,63,64], neurodegenerative diseases [61,65] and other diseases with relevant pathologies. Research has yet to turn to loading small molecules in stem cell-derived EVs, specifically for tissue engineering and regenerative medicine, but several studies have nonetheless demonstrated the possible appeal and feasibility of using stem cell-derived EVs as a drug delivery vehicle. Much of small molecule loading has used passive methods of encapsulation, with active loading methods being less common techniques. Cell preconditioning or priming, for example, has been an effective form of loading drugs within EVs. MSC-derived EVs, in particular, have been used in cancer therapy due to the ability of MSCs to preferentially target tumor sites. Paclitaxel, a popular and effective anticancer drug, can be uptaken in significant amounts by bone-marrow MSCs in culture and then be subsequently released within EVs, as a form of passive encapsulation [64,66]. Isolated EVs collected from the primed MSCs showed significant anti-proliferative effects on a human pancreatic cell line, suggesting successful encapsulation of paclitaxel. The ability of cells to uptake and release small molecule drugs via EVs is not only unique to bone-marrow MSCs, but are also found in other stromal cells isolated from adipose tissue [67] and the derma [68]. Incubation and simple mixing have been shown to be another successful method by which small molecules have been loaded into EVs. EL-4-derived EVs were mixed with curcumin at 22 °C, with a loading efficiency of 2.9 g of curcumin to 1 g of EVs [60]. The anti-inflammatory properties of the curcumin-EVs were assessed in a septic shock murine model, where the curcumin-EVs significantly reduced levels of IL-6 and TNF-α and increased mice survival.

#### 3.1.2. Nucleic Acids

Throughout the field, the loading of nuclei acids, such as mRNAs, microRNAs (miRNAs) and small interfering RNA (siRNA), within EVs for tissue regeneration has been heavily favored, possibly due to their natural presence as EV cargo. Though much of current EV engineering research for the active encapsulation of nucleic acids has been mainly applied for cancer therapies, these studies have nonetheless set a precedent for the similar use of engineered EVs for regenerative medicine. B-cell-derived EVs were loaded with exogenous miRNA-155 inhibitors and delivered to target macrophages as a gene therapy strategy to modulate inflammatory activation [69]. Functional miR-155 mimics were electroporated into the EVs and delivered intravenously to miR-155 knockout mice, where there was found to be a statistically significant increase in miR-155 levels in primary hepatocytes in the liver. These miR-155 inhibitors were similarly electroporated into EVs and cultured in vitro with RAW 264.7 macrophages. After stimulation with lipopolysaccharide, levels of TNF-α, a potent marker of inflammation, were significantly diminished as well as the expression of endogenous miRNA-155. SOCS1 mRNA, a cytokine suppressor gene, was significantly increased, indicating the ability of the engineered EVs to regulate expression of anti-inflammatory genes. EVs can also be engineered for nucleic acid delivery at a cellular level using preconditioning or exogenous transfection to induce the enrichment of natural miRNAs in EVs. Cell preconditioning has also been validated as a method of enriching key miRNAs. Cardiac progenitor cells were stress-treated via H_2_O_2_ incubation to produce miR-21 enriched EVs that were used to protect cardiomyocytes from damage in ischemic conditions [70]. Hypoxic preconditioning of cells has also shown to be a successful method of engineering miRNA expression in EVs. Next generation sequencing revealed that EVs originating from human neural stem cells cultured under hypoxic conditions have increased levels of 53 miRNAs and decreased levels of 26 miRNAs [71]. 

Microarray analysis of EVs secreted by hypoxic cardiac progenitor cells showed that 11 miRNAs were upregulated at least 2-fold compared to EVs secreted from normoxic cells [72]. However, preconditioning offers limited control in the specific types of miRNA that is enriched, as only naturally-occurring cargo can be overexpressed. Additionally, standardization remains a problem, as different conditions can lead to different types and levels of cargo enrichment. Transfection or transduction, though still an indirect method of cargo loading compared to electroporation or other active methods for exogenous cargo loading, offers more control than preconditioning. Umbilical cord MSCs were transfected with miR-675 mimic, leading to the secretion of EVs that showed increased expression of miR-675 [73]. When tested in a murine model for aging-induced vascular dysfunction, the miR-675-enriched EVs, delivered in silk fibroin hydrogels, successfully promoted blood perfusion in ischemic hindlimbs and reduced the expression of proinflammatory molecules. Similarly, adipose tissue-derived MSCs were transfected with plasmids expressing miR-122, thus producing EVs enriched in miR122 that were then used successfully as a chemotherapeutic agent for hepatocellular carcinoma [74]. Lentiviral transduction has also been successful in genetically engineering MSCs to overexpress miR-let7c and secrete EVs enriched in miR-let7c [75]. These enriched EVs were seen to mediate the improved transfer of miR-let7c to target the kidneys and attenuate renal fibrosis.

siRNAs are another class of small RNAs which have been exogenously loaded into EVs. siRNA loading is similar to that of miRNA loading, with electroporation being the most common method of active loading [76]. But unlike miRNAs, siRNAs have also been loaded via conjugation of siRNA molecules to cholesterol tags. In a study conducted by Stremersch et al., siRNA was conjugated to cholesterol anchors (chol-siRNA), which enabled the self-insertion into the lipid outer vesicular membrane of B17F10 melanoma- and JAWSII monocyte-derived EVs [77]. Binding of the siRNA to the EV outer membrane was successful, with about 73 chol-siRNA molecules per vesicle, and was able to be successfully trafficked to target cells. However, subsequent functional studies revealed that there was no significant knockdown effect of target gene expression even though chol-siRNA only without the nanocarriers did demonstrate a knockdown effect. The functional impairment may be due to the immobilization of the siRNA to the EV surface, which may hinder siRNA activation of RNAi mechanisms. However, O’Loughlin et al. showed that the cholesterol-siRNA (cc-siRNA) could be induced to be loaded within the vesicle under optimal parameters (15 molecules of cc-siRNA per EV at 37 °C for 1 h in 100 μL) [78]. Incubating at higher temperature allows for greater membrane fluidity, thus allowing for the greater interaction between EVs and the cc-siRNA, and ultimately leading to greater internalization and encapsulation. This temperature dependency may explain why Stremersch et al. observed chol-siRNA conjugation on the membrane surface and no functional effects after incubation at room temperature.

#### 3.1.3. Proteins

The loading of exogenous proteins into EVs has been less studied compared to its nucleic acid and small molecule counterparts, but is nevertheless a viable area of study. EVs are known to shuttle a variety of functional proteins to target cells as part of their mechanism of action. Proteins can be loaded in EVs using a WW domain tag, which is known to bind Ndfip1, an ubiquitin ligase adaptor protein, that has been confirmed to be exported from the cell in EVs [79]. Therefore, by modifying a protein of interest, in this case a recombinase protein known as Cre, with the WW tag, the protein can be bound to the Ndfip1, which in turn can guide the modified target protein into EVs. This EV engineering was indirect, as the parent cell was modified rather than the EVs directly. A plasmid was coded for the WW–Cre fusion protein and transfected into LN18 and JEK293T cells [80]. Loading into EVs was confirmed in the presence of Ndfip1 using a WW-mCherry fluorescent reporter, while no loading occurred without Ndfip1. Targeting of the plasma membrane anchors has been similarly proven to be a strategy for preferentially sorting proteins into EVs before vesicle budding. Engineering a myristoylation tag or targeting phosphatidylinositol-(4,5)-bisphosphate (PIP2), all showed highly successful budding of a model TyA-GFP protein in EVs [81]. Using a prenylation/palmitoylation tag or targeting the CD43 or phosphatidylinositol-(3,4,5)-trisphosphate-binding domain were less efficient in facilitating TyA-GFP budding, but nevertheless induced some level of budding [81].

Haney et al. demonstrated more direct ways of loading proteins into EVs using a variety of methods: coincubation, chemical permeabilization of the membrane, freeze –thaw cycles, sonication and extrusion [65]. Using catalase as the loading protein, extrusion and sonication proved to be the most efficient loading techniques, followed by freeze–thaw cycles, and then simple incubation at room temperature. However, the loading efficiency for even the most successful sonication and extrusion techniques was about 26% and 22%, respectively, still a relatively low loading capacity. Nevertheless, the catalase function was importantly conserved following EV loading, shown by neuroprotective effects in the in vitro and in vivo murine models of Parkinson’s disease. In comparison, free catalase was deactivated and lost its enzymatic functions in the same media conditions.

**Table 1:** Comparison of loading mechanisms of different cargo into extracellular vesicles (EVs).

Method	Description	Loading Type	Cargo
**Incubation**	EVs and cargo are mixed together and coincubated. Cargo molecules follow the concentration gradient to diffuse into EVs.	Passive	Protein [65] Small molecules [60,62]
**Cell preconditioning or engineering**	Cells are cultured in specific environmental conditions, or are transfected/transduced to induce the secretion of cargo-enriched EVs.	Passive	Small molecules [64,67,68] miRNA [70,73,74,75] Protein [79]
**Freeze/thaw cycle**	EV and cargo molecules are incubated together at room temperature and then rapidly frozen at <−80°C and thawed at room temperature. The freezing disrupts the membrane, which allows the cargo molecules to diffuse into the EVs while in a semi-frozen state during the thawing process.	Active	Protein [65]
**Sonication**	A sonicator probe is used to create a mechanical shear force which deforms the EV membrane structure to allow the cargo molecules to diffuse into the EV.	Active	Protein [65] siRNA [82]
**Extrusion**	EVs and cargo molecules are loaded into a syringe and injected forcefully though a porous membrane. The mechanical force disrupts the EV membrane and entraps the cargo within the EV.	Active	Small molecule [83] Protein [65]
**Chemical permeabilization**	Surfactants, or other similar molecules, complex with cholesterol on EV membranes to generate pores, through which cargo molecules can permeate through the EV membrane.	Active	Small molecule [83] Protein [65]
**Electroporation**	EVs are mixed in a solution containing cargo molecules. A voltage is applied that creates temporary pores in the EV membrane which allows these cargo molecules to diffuse into the EVs. When the voltage is removed, the membrane reseals and traps the cargo within the EV.	Active	Small molecule [83,84] siRNA [76] miRNA [69]

### 3.2. Surface Modification

Native EVs express cell adhesion molecules and ligands as a result of budding from cell plasma membranes that endow these vesicles with a variety of natural targeting specificities. For example, MSC-derived EVs express key regulatory ligands such as PDL1, TGF-β and galectin-1 that allow for specific uptake by target T-cells as a mechanism by which autoreactive CD4 lymphocytes found in an experimental autoimmune encephalitis (EAE) phenotype are attenuated [85]. Neuroblastoma EVs, in turn, are highly enriched in glycosphingolipids that target and eliminate amyloid-β peptide, a protein that is involved in the pathogenesis of Alzheimer’s disease, and its metabolites [86]. The natural targeting ligands on these EVs have inspired research into synthetic ligand binding to vesicles to improve target specificity. Several binding mechanisms have been established for EV surface modification. 

#### 3.2.1. Click Chemistry

Click chemistry, also known as an azide alkyne cycloaddition, is a method by which molecules can be directly attached to EV surfaces via covalent bonds [87,88]. In this reaction, an alkyne moiety is reacted with an azide group to form a stable triazole linkage. This reaction is greatly accelerated with the use of a copper catalyst [87,89], but several studies have also demonstrated successful binding using copper-free click chemistry [90]. Click chemistry has emerged as a popular method of bioconjugation due to its relatively mild experimental conditions which generally involve a variety of solvents such as water, alcohols and dimethyl sulfoxide (DMSO), as well as its high yield and simplicity [88]. Additionally, the azide functional group is absent in natural biomacromolecules, thus eliminating the possibility of off-target binding [88]. Use of Click chemistry on EVs was validated in a study by Smyth et al., who reported that an azide-flour 545 could be successfully conjugated onto EV surfaces using Click chemistry [91]. EVs were first modified with alkynes using *n*-hydroxysuccinimide (NHS) and pentynoic acid and underwent Click chemistry when reacted with azide-fluor 545 in the presence of copper and salts. The triazole linkage that resulted finally facilitates the azide-fluor 545 binding to the EV surface. This conjugation was found to have no impact on EV size and did not hinder or otherwise negatively impact target cell uptake. However, the critical alkyne modification of the EV surface most likely occurs on EV proteins rather than the amines on the membrane phospholipids. As such, this introduces the possibility that the EV protein function may be partially or fully inhibited by this modification. Nevertheless, the successful binding introduces a proof-of-concept that allows for functional peptide binding to EV surfaces. 

This possibility was further realized when Jia et al. used Click chemistry to conjugate the neuropilin-1 (NRP-1)-targeted peptide, RGE, to the surface of macrophage-derived EVs [92]. EV membranes were modified with sulfo-NHS and then reacted with azide-modified RGE peptide using salts and copper as catalysts. Successful conjugation was validated by the use of fluorescein isothiocyanate (FITC) azido-RGE using fluorescence microscopy.

#### 3.2.2. Integrin-Binding

Following their budding off from the plasma membranes, EVs retain many membrane proteins, some of which are EV-specific, that offer a potentially new method for EV surface modification. Lamp2b, a membrane protein found on EVs, has been a popular target for fusing targeting peptides. In one instance, cardiac-targeting peptide (CTP) was expressed on the surface of human embryonic kidney cell (HEK)-derived EVs by fusing the CTP peptide to the N-terminus of the Lamp2b via plasmids [93]. The resulting CTP-EVs were seen to preferentially target cardiac cells in vitro (16% increase) and in vivo in a mouse model (15% increase) when compared to control scrambled-peptide-modified EVs. Similar methods with the Lamp2b protein were used to fuse the RVG and MSP peptides to target murine neuronal and muscle cells, respectively [76]. However, the efficiency of Lamp2b protein tagging was greatly diminished due to lysosome-mediated proteolytic degradation [94]. As a way to protect peptide degradation and improve function, glycosylation motifs were engineered onto the EV surfaces by using plasmid transfections to fuse N-glycosylation sequences to the N-terminus of the Lamp2b, which, in turn, has already been bound with the targeting peptide [94]. The glycosylation tags protected the targeting peptide from proteolysis without inhibiting the targeting capabilities, and this further enhanced EV uptake by recipient cells.

More typical transmembrane glycoproteins (e.g., α3β1, α4β1 and αvβ3) have also been a target for peptide binding. Carney et al. used the one-bead one-compound (OBOC) combinatorial library approach to identify the LXY30 ligand which was shown to specifically bind to the α3β1 integrin [95]. This specificity allowed conjugation to EVs derived from ovarian tumor cells, which have a high expression of α3β1. Flow cytometry revealed a high binding of LXY30 to the EVs, while scrambled-LXY30 did not measurably bind to EVs, indicating a high specificity binding affinity. Different ligands also identified through OBOC technology have similar binding affinities to other integrins, such as LXW7 for αvβ3 [96] and LLP2A for α4β1 [97]. Though these ligands have not been reported to confer any specific targeting properties to EVs, they can nonetheless be used as a diagnostic tool to identify EV types or be used as linkers to associate more functional molecules to EVs.

#### 3.2.3. Phospholipid-Domain Binding

EVs are enriched in phosphatidylserine (PS), which allows for significant opportunity for surface modification. Kooijmans et al. fused PS-targeting protein C1C2 with ligands for epidermal growth factor receptors (EGFR) [98] to generate recombinant fusion protein-encoded vectors that were transfected into HEK293 cells. The produced protein was isolated and successfully self-associated with PS on the outer membrane of red blood cell-derived EVs. Uptake studies showed a decreased uptake of modified EVs by non-targeted cells, while significant uptake was seen in EGFR-expressing cells, suggesting minimal off-target delivery. A similar approach was used to functionalize L929-derived EVs with a proaptotic peptide KLA and low-density lipoprotein (LDL) using L-4F, an ApoA-I mimetic peptide [99]. L-4F preferentially binds to the phospholipid-rich regions of EVs, therefore acting as a linker to conjugate the functional peptides to the EV. Successful conjugation and subsequent functionality were confirmed with the increased permeation of EVs through the blood–brain barrier and penetration into glioma spheroids. The phospholipid-binding method has also been shown to be successful using a more passive approach. Dual ligands, biotin and avidin, were conjugated to the plasma membrane of parent cells using a distearoyl-sn-glycero-3-phosphoethanolamine-poly(ethylene glycol) (DSPE-PEG) linker, where the DSPE end self-inserts into the phospholipid membrane and the PEG end binds the ligand [100]. When the engineered cells secreted EVs through fusion of the plasma membrane, the EVs were found to have retained the ligands on their membrane surface.

### 3.3. EV Hybrids

Hybridization of EVs with their synthetic counterparts, liposomes, has been another approach used to optimize and augment the properties of natural EVs. The lipid composition of EV membranes has made it an excellent candidate for membrane fusion with artificial liposomes. This method improves the colloidal stability of EVs, thus increasing its half-life in blood and decreasing its immunogenicity [101]. Sato et al. synthesized hybrid EV/liposomes using a freeze–thaw method, where EV/liposome mixtures are frozen in liquid nitrogen and then thawed at room temperature [101]. Fluorescence resonance energy transfer (FRET) quantitatively verified the high efficiency of EV and liposome fusion, while transmission electron microscopy (TEM) could not find any apparent membrane morphological differences after membrane fusion. Interestingly, the membrane fusion efficiency varies among EV types, as those derived from CMS7-HE (CMS7 cells overexpressing the HER2 receptor) have a relatively higher fusion efficiency than those isolated from RAW 264.7 cells. This may be due to the variability in membrane lipid composition and protein content between EVs, thus impacting interaction with liposomes, and in turn, fusion efficiency.

Lipid composition is also seen to impact cellular uptake. EVs hybridized with cationic lipids have lower cellular uptake efficiencies while neutral and anionic lipids do not have any observable effects. However, the addition of molecular chains such as polyethylene glycol (PEG) onto the engineered EV surfaces greatly increases cellular uptake, possibly due to the PEG reducing the electrostatic repulsions between the EV membrane and the target cell membrane. Hybridization of EVs additionally increases the size of the vesicles [101,102]. The magnitude of the size increase, however, may be technique dependent. The freeze–thaw method is seen to lead to anywhere between a 40–100 nm increase in size [101], while the passive incubation of EVs and liposomes after 12 h at 37 °C causes over a 400 nm increase in average size [102]. While the size increase may decrease the in vivo retention of the nanoparticles [103], it is advantageous in that it can improve the drug encapsulation efficiency. Native EVs are limited in their ability to encapsulate large nucleic acids due to their small size, while the larger hybridized EVs can carry larger cargo, such as plasmids for CRISPR/Cas9 systems [102].

## 4. Synthetic EVs

Despite their great therapeutic potential, EVs unfortunately come with a variety of problems that make clinical translation quite difficult. One main problem is the difficulty in isolating a large EV yield. The current gold standard for isolating EVs involves long culture times to obtain hundreds of millions of cells and intensive ultracentrifugation steps, all of which are expensive and require a great deal of time. Additionally, EV properties can vary between cell batches and passage number, making standardized therapies difficult to manufacture. While quality control and good manufacturing practices can improve EV standardization, there still remains a degree of heterogeneity that is difficult to eliminate. As a potential solution to improve the clinical translation of EVs, another side of research has focused solely on the synthesis of EV-mimics, made of natural or artificial materials, to recapitulate the functions of native EVs while eliminating their inherent disadvantages. This would improve the clinical translation of EVs, as engineered vesicles are relatively easier to synthesize on a larger scale and with more homogeneity due to greater control and optimization over the synthesis procedures. The major techniques involving the synthesis of artificial EVs can be classified into either the top-down or bottom-up approaches. These different techniques have been summarized in Table 2.

### 4.1. Top-Down Technique

The top-down approach begins with using a bulk material from which nanosized units are derived. With this technique, multiple larger components or features are reduced to form the desired biomaterial. An excellent example of this is that of the EV-like nanovesicles derived from whole cell fragments. This approach relies on the idea that EVs originate from the endogenous compartments and plasma membrane of the cell.

#### Cell-Derived Nanovesicles

**Figure 2:**
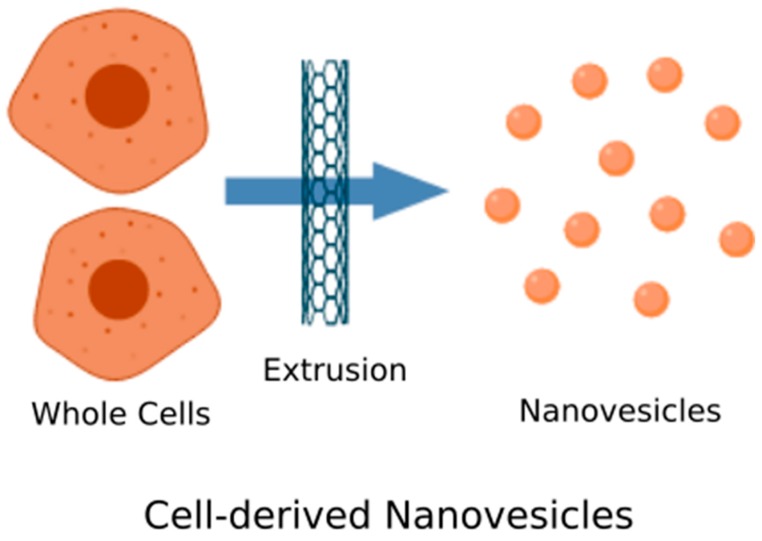
Cell-derived nanovesicles. Whole cells are mechanically extruded to break the cell and create plasma membrane fragments. The fragments then self-assemble into nanovesicles that can retain intracellular molecules and surface makers.

EV mimics have also been synthesized by fragmenting whole cells until small membranous vesicles are formed (Figure 2). Membrane fragmentation can be done in several different techniques, but one common method is using serial extrusion through polycarbonate filters of decreasing pore sizes. Jang et al. synthesized EV-mimicking nanovesicles by extruding human monocytic cells through filters with pore sizes of 10, 5 and 1 μm [104]. The resulting nanovesicles were between 120–130 nm in size, similar to native monocytic EVs, and expressed multiple EV marker proteins. In vitro and in vivo tests of functional properties showed that these synthetic nanovesicles loaded with the well-known cancer drug, doxorubicin, had comparable targeting specificity and antitumor effects as native monocytic EVs. As such, it is especially remarkable when authors showed that synthetic EVs were able to be produced at about a 100-fold greater yield than native EVs secreted by the same number of cultured cells.

Similar methods were used to synthesize EV-mimicking nanovesicles from pancreatic β-cells in which cells were harvested and serially extruded through 10, 5 and 1 μm pore-sized polycarbonate membrane filters [105]. Assessment of the physical properties of nanovesicles revealed an average size of 203 nm, and it has been found that protein contents similar to donor cells though insulin and Pdx1, a transcription factor that regulates β-cell development, were not detected. Regardless, functional studies showed that the nanovesicles directed bone marrow cell differentiation into insulin-producing cells in vitro, that when transplanted into diabetic mice, were capable of controlling blood glucose levels for 60 days. Though no control comparison was made to native EVs derived from the same β-cell line, a yield of 8.2 × 10^8^ particles per 1 μg of total protein was reported, confirming that generally a high number of vesicles can be made from this method [105]. A similar protocol was used to produce EV mimics from human breast epithelial cells [106]. A significantly higher yield of mimics was seen, with 379.3 μg of protein and 3.0 × 10^11^ of mimics produced in comparison to 2.5 μg of protein and 2.6 × 10^9^ of EVs from the same number of cells (1.0 × 10^7^ cells). EV-specific surface markers were conserved in the mimics, while functional assessments revealed that the mimics did not stimulate any immune response in mice, and exhibited excellent targeting capabilities to tumor cells.

Large-scale generation of EV-mimicking nanovesicles can also be made by centrifuging cell suspensions through a microporous polycarbonate membrane enclosed in a larger polycarbonate structure designed to fit in centrifuge bucket holders [107]. The highest yield rate was found to be at ~16,000 kPa (2000 rpm) using a 1 × 10^8^ murine embryonic stem cell (ES) suspension, which led to about 250 times more nanovesicles than the EVs produced from an equal number of ES cells. Western blotting and a reverse transcription polymerase chain reaction (RT-PCR) of both nanovesicles and EVs showed remarkable similarities in protein expression and RNA profiles while size characterization revealed similar ~100 nm diameters [107].

Another method of generating nanovesicles involves using a microfluidic system. Live murine embryonic stem cells were extruded through microchannels lined with an array of 500 nm-thick silicon nitride blades that were used to slice pieces of plasma membrane from the cells [108]. The plasma membrane pieces spontaneously self-assembled into nanovesicles that retained cellular contents (proteins, intracellular RNAs) or was loaded with exogenous molecules. Resulting nanovesicles were 100–300 nm in size and had a yield of ~150 × 10^8^ from 1 × 10^6^ cells.

### 4.2. Bottom-Up Technique

The bottom-up process is much more specific in that the nanoscale components are carefully chosen and combined to form the larger and more complex structure. The bottom-up design allows for the creation of tailored structures that can address a specific purpose. However, they can be more difficult to synthesize, as piecing together different components may involve complex binding techniques and create new challenges.

#### 4.2.1. Liposomes

Liposomes are a well-studied drug delivery system that has had tremendous success for a variety of clinical applications, from cancer treatments (i.e., Doxil^®^, Myocet^®^, Marqibo^®^) to viral vaccines (i.e., Expaxa^®^, Inflexal^®^ V) to fungal diseases (i.e., Abelcet^®^, Ambisome^®^, Amphotec^®^) [109]. Indeed, considering their characteristics, which namely are that of a lipid bilayer with a wide range of cargo-carrying possibilities, and their targeting potential, liposomes can be considered the artificial counterparts to EVs. Their lipid bilayer composition makes liposomes an excellent mimic, as EVs are similarly composed of a variety of lipids, specifically phospholipids, such as phosphatidylcholine (PC), phosphatidylethanolamine (PE) and sphingomyelin [110]. Lu et al. synthesized EV-mimicking liposomes using a lipid formulation comparable to the EV lipidomic profile [111]. Liposomes were created using a DOPC/SM/Chol/DOPS/DOPE mixture and loaded with VEGF siRNA to model EV cargo. Compared to conventional PC/Chol and DOTAP liposomes, the EV-mimicking liposomes enhanced cellular uptake, serum stability and siRNA silencing, though there was lower encapsulation efficiency. Analogous phospholipid-based liposomes have been synthesized, to great effect, for regenerative applications [112].

**Figure 3:**
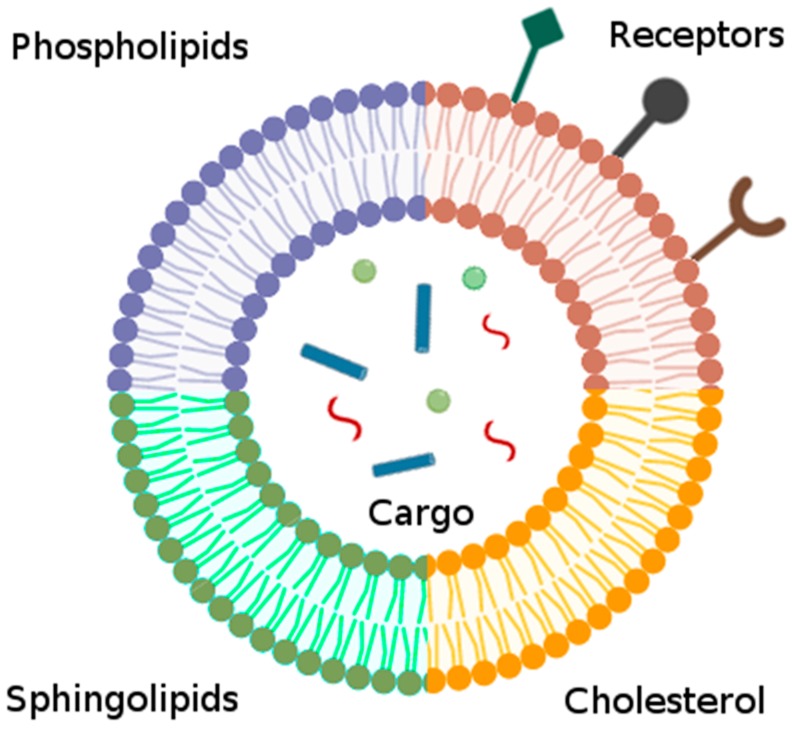
Extracellular vesicle (EV)-mimicking liposomes can be engineered to mimic EV features by recreating the lipidomic profile (e.g., phospholipids, sphingolipids, cholesterol), and by conjugating proteins and receptors to recreate the targeting specificity. The liposomes can also be loaded with a variety of molecules, including proteins, siRNAs, miRNAs and small molecules, to recapitulate common EV cargo.

To further mimic EVs, liposomes can also be modified with targeting peptides to improve targeting specificity and function (Figure 3). Common peptides include various derivatives of ligands for integrins (e.g., αvβ3 and αvβ5), G-protein coupled receptors and growth factor receptors. Zhang et al. developed artificial chimeric exosomes (ACEs) by embedding cell membrane proteins derived from red blood cells and MCF-7 breast cancer cells, to evade the immune system and facilitate adherence to homologous cancer cells, respectively, into the phospholipid bilayers of liposomes [113]. The integration of the cell membrane proteins mimicked the morphological and physiological composition of EVs, while functional abilities were recapitulated in the improved targeting ability and anti-phagocytosis capabilities. Broadly speaking, conjugation of these peptides to liposome surfaces showed significant selectivity and uptake by target cells while having no significant cytotoxic effects, much like EVs. Peptide expression further plays a role in improving the in vivo retention rate, a property that has long hindered liposome treatments, but is a strength found in native EVs. 

Despite many similarities, and regardless of countless engineering strategies, liposomes are still far from perfect mimics of EVs due to their low stability, retention and lack of immunomodulatory properties [114]. 

#### 4.2.2. Biomimetic Polymer Nanoparticles

**Figure 4:**
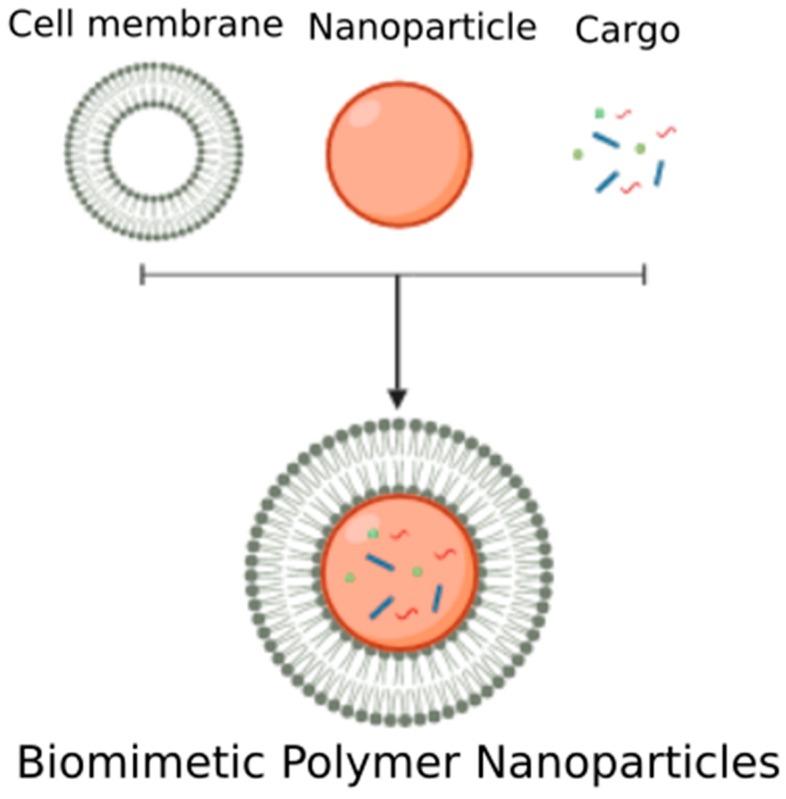
Biomimetic Polymer Nanoparticles. Cell plasma membrane that has been isolated and purified can be mixed with a polymer-based nanoparticle to create a cell-membrane-cloaked particle. Different types of cargo can be loaded within the polymer core.

Polymeric biomaterials offer a stability not found in classic liposome structures, but lack the biocompatibility and biological structure that could mimic EVs. To combat this, a new class of biologically-inspired polymeric nanoparticles have emerged, one which can arguably mimic EVs. In this system, a polymeric core is created to provide structural support and stability, while a biological material, such as cell plasma membrane, is used to coat the polymer core surface to provide a biocompatible shell (Figure 4). One of the first instances of a cell membrane coating on a polymer core is by Hu et al., who coated sub-100 nm poly(lactic-co-glycolic acid) (PLGA) nanoparticles with red blood cell (RBC)-membrane-derived vesicles [115]. Though the intention of this system was to develop a long-circulating drug delivery system, it resulted in an excellent EV mimic. The RBC-membrane-coated nanoparticles could be successfully loaded with proteins in the stable polymer core, much like EVs carry protein cargo. Furthermore, the RBC membrane coating retained the biological proteins and contributed to the overall high stability and immune evasion capabilities of the nanoparticle, again similar to the immunomodulatory properties of EVs. Umbilical cord-derived MSC membrane-coated PLGA nanoparticles demonstrated similar stability, improved cellular uptake, targeting and immune evading characteristics that are similarly seen to be exhibited by EVs in literature [116]. Subsequent studies have analyzed more of the biological mimicking properties of these hybrid polymer systems. Parodi et al. found that functionalizing nanoporous silicon particles with leukocyte cell membranes imparts on the system cell-like properties [117]. These hybrid particles, termed leukolike vectors, successfully performed multiple leukocyte functions, including communicating with endothelial cells via receptor-ligand interactions, delivering payload to cells (simulated by doxorubicin encapsulation), specific targeting ability and improved circulation times.

However, the biological functions are seen to be dependent on the origin of the cell membrane. When PLGA cores are coated with cell membranes derived from either mesenchymal stem cells or cardiac stem cells, the resulting biomimetic particles exhibited functional properties unique to cell type, such as promoting the paracrine secretion of growth factors, or augmenting cardiac functions, respectively [118,119]. This suggests that the cell membrane coating provides important and specific biological and functional properties, while the polymer core allows for the encapsulation of a variety of target-specific cargo.

**Table 2:** Summary of semi-synthetic and synthetic EV-mimic types.

Synthetic EV Type	Description	Method
**Native EV Modification**	Native EVs can be engineered to improve their drug loading, targeting and yield.	Drug loading [60,61,62,63,64,75]
		Surface peptide modification [91,92,98,99]
		Liposome hybridization [101,102]
**Nanovesicles**	Whole cells are fragmented into membrane pieces that self-assemble into vesicles.	Serial extrusion [104,105,106,107]
		Microfluidics [108]
**Liposomes**	Bilayered, spherical lipid vesicles that can be loaded with cargo or modified with surface proteins mimicking native membrane proteins.	Surface-conjugated peptides [113]
		Tailored lipid formulation [111,112]
**Biomimetic Polymer Nanoparticles**	Polymer-based nanoparticles are created and mechanically coated with plasma membrane to create nanovesicles with a core-shell structures.	PLGA core, plasma membrane coating [115,116,120,121]
		Silicon core, plasma membrane coating [117]

## 5. Future Perspectives

EVs have remarkable biological functions that make them an incredible therapeutic option. Their uses span across treatments for diseases such as cancer to promoting tissue regeneration in regenerative medicine. As of 2019, there are 148 EV-related clinical trials in the United States reported by clinicaltrials.gov, indicating that there is widespread interest in using EVs as therapeutics for clinical applications. As such, EVs have started to be evaluated in clinical trials over the past decade, though they still have not yet been approved. Considering the myriad of EV isolation, purification and standardization procedures, the regulatory framework regarding EV therapeutics is very much nascent. Currently under current European Union (EU) and United States (US) statutes, EVs are regulated as drugs and biological products [34]. To meet proper approval procedures, it is critical that all steps of the EV isolation process are cGMP-compliant. Cell lines must be thoroughly checked to determine whether they adhere to eligible donor criteria, such as screening for infectious diseases and strictly evaluating donor medical history [31,34]. Culture conditions and EV isolation procedures must be carefully standardized to prevent any confounding factors from affecting EV production and reproducibility. This is especially key when scaling up the EV production as downstream processing must still remain consistent. Following isolation, EVs have to be assessed for functionality through the use of a standardized potency assay in order to account for any variations between EV batches [34]. Initial safety studies must also be conducted with early stage trials before moving onto larger scale, human clinical studies, and ultimately to market.

Currently, difficult isolation procedures, low yield and functional heterogeneity, are major roadblocks that continue to hinder clinical application. Therefore, the field has turned to engineering EVs to enhance their therapeutic functions. The advent and success of synthetic EVs offer a hopeful alternative in which the extremely advantageous biological properties of EVs can be harnessed in a more homogenous, larger-scale artificial system. Considering the countless biomaterials options, the adaptable and highly tailorable properties of synthetic EVs additionally offer future applications for personalized medicine, where unique systems can be synthesized to target specific diseases on an individual basis. Nevertheless, significant challenges remain before synthetic EVs can be a viable reality. Much research remains to be done on the reproducibility of synthetic EVs, both on a functional and physical level, as well to confirm the safety and regulatory protocols for large-scale manufacturing and clinical translation. With continuing research and evolving technology, synthetic EVs could be the next step towards personalized medicine.
